# Green Biosynthesis of Silver Nanoparticles Using *Callicarpa maingayi* Stem Bark Extraction

**DOI:** 10.3390/molecules17078506

**Published:** 2012-07-16

**Authors:** Kamyar Shameli, Mansor Bin Ahmad, Emad A. Jaffar Al-Mulla, Nor Azowa Ibrahim, Parvaneh Shabanzadeh, Abdolhossein Rustaiyan, Yadollah Abdollahi, Samira Bagheri, Sanaz Abdolmohammadi, Muhammad Sani Usman, Mohammed Zidan

**Affiliations:** 1 Department of Chemistry, Faculty of Science, Universiti Putra Malaysia, Serdang UPM 43400, Selangor, Malaysia; Email: mansorahmad@gmail.com (M.B.A.); emadaalmulla@yahoo.com (E.A.J.A.-M.); norazowa@science.upm.edu.my (N.A.I.); s.abdolmohammadi@yahoo.com (S.A.); muhusma@yahoo.com (M.S.U.); zidanupm@gmail.com (M.Z.); 2 Materials and Energy Research Center, Karaj 317-798-3634, Iran; 3 Department of Chemistry, College of Science, University of Kufa, B.O. Box 21, An-Najaf 54001, Iraq; 4 Department of Chemical Engineering, Faculty of Engineering, Islamic Azad University, Malard Branch 316-915-3174, Iran; Email: parvaneh.shabanzade@gmail.com; 5 Department of Chemistry, Science and Research Branch, Islamic Azad University, Tehran 145-157-75, Iran; Email: arustaiyan@yahoo.it; 6 Advance Materials and Nanotechnology Laboratory, Institute of Malaysia Advance Technology, Serdang UPM 43400, Selangor, Malaysia; Email: yadollahabdollahi@yahoo.com; 7 Centre of Research in Nanotechnology and Catalysis (COMBICAT), IPS Building, University of Malaya, Kuala Lumpur 50603, Malaysia; Email: samira6363@yahoo.com

**Keywords:** biosynthesis, silver nanoparticles, *Callicarpa maingayi*, green synthesis, fourier transform infrared

## Abstract

Different biological methods are gaining recognition for the production of silver nanoparticles (Ag-NPs) due to their multiple applications. The use of plants in the green synthesis of nanoparticles emerges as a cost effective and eco-friendly approach. In this study the green biosynthesis of silver nanoparticles using *Callicarpa maingayi* stem bark extract has been reported. Characterizations of nanoparticles were done using different methods, which include; ultraviolet-visible spectroscopy (UV-Vis), powder X-ray diffraction (XRD), transmission electron microscopy (TEM), scanning electron microscopy (SEM), energy dispersive X-ray fluorescence (EDXF) spectrometry, zeta potential measurements and Fourier transform infrared (FT-IR) spectroscopy. UV-visible spectrum of the aqueous medium containing silver nanoparticles showed absorption peak at around 456 nm. The TEM study showed that mean diameter and standard deviation for the formation of silver nanoparticles were 12.40 ± 3.27 nm. The XRD study showed that the particles are crystalline in nature, with a face centered cubic (fcc) structure. The most needed outcome of this work will be the development of value added products from *Callicarpa maingayi *for biomedical and nanotechnology based industries.

## 1. Introduction

Nanoparticles are normally considered as particles with a maximum size of 100 nm. They display completely novel or improved properties, which are quite different from those of larger particles. In the compared to the larger particles of the bulk material that they are composed of based on specific characteristics such as size, shape, distribution, and surface morphology [1]. Metal nanoparticles, such as Ag, Au, Pt and Pd, are extensively applied in products that directly come in contact with the human body, such as household items like detergents, soaps, shampoos, cosmetic products, and toothpaste, and they also find applications in the pharmaceutical and medical area [2].

Silver nanoparticles (Ag-NPs) have definite properties. This may perhaps have numerous applications in the fields of dentistry, clothing, catalysis, mirrors, optics, photography, electronics and food industries [3]. Because of such broad variety of applications, wide ranges of different preparation methods have been developed. However, the developing methods of Ag-NPs preparation, must give preference to control size of Ag-NPs. Therefore, nanosilver with small particle size and devoid of aggregation between particles is favorable in this purpose.

There are several ways to reduce Ag^+^: Use of γ-rays [4], UV irradiation [5], heating and electrochemical reduction [6], application of reducing chemicals, such as hydrazine [7], sodium borohydride [8,9,10], polyethylene glycerol [11], *N,N*-dimethylformamide [12], glucose [13], ethylene glycol [14], formaldehyde [15], sodium in liquid ammonia, *etc.* [16]. However, there is still need for a more economic, commercially viable as well environmentally green synthesis route to synthesize Ag-NPs. The green synthesis of Ag-NPs involves three main steps, which must be evaluated based on green chemistry perspectives, including selection of solvent medium, reducing agent and nontoxic stabilizers for Ag-NPs [17].

The biosynthesis of nanoparticles, which represents a connection between biotechnology and nanotechnology, has received increasing consideration due to the growing need to develop environmentally friendly technologies for material syntheses. The search for appropriate biomaterials for the biosynthesis of nanoparticles continues through many different synthetic methods [18].

The biosynthetic method using plant extracts has received more attention than chemical and physical methods and even than the use of microbes. The method is suitable for nanoscale metal synthesis due to the absence of any requirement to maintain an aseptic environment [19]. The possibility of using plant materials for the synthesis of nanoscale metals was reported initially by Gardea-Torresdey *et al*. [20,21]. Later, the bioreduction of various metals to nanosize materials of various shapes, capable of meeting the requirements of diverse industrial applications, was extensively studied [22]. In continuation, Zargar *et al*. demonstrated the prospect of using *Vitex Negundo L*. leaf methanolic extract for the synthesis of the Ag-NPs at ambient conditions, without any additive protecting nanoparticles from aggregating, template shaping nanoparticles or accelerants [23].

In this study, the synthesis and characterization of Ag/*Callicarpa maingayi* by a green method is reported. The Ag-NPs were prepared using silver nitrate as silver precursor and *Callicarpa maingayi *stem bark methanolic extract as reducing agent and stabilizer.

## 2. Results and Discussion

**Figure 1:**
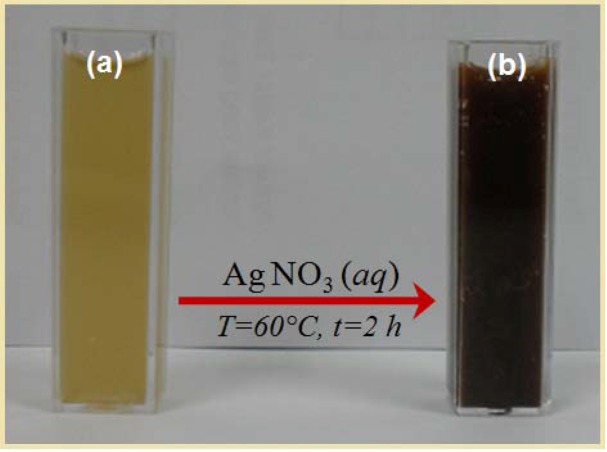
Photograph of (**a**) *Callicarpa maingayi* and (**b**) Ag/*Callicarpa maingayi* emulsion after 48 h.

Reduction of Ag^+^ into Ag-NPs during exposure to *Callicarpa maingayi *stem bark extracts could be followed by the colour change. The fresh suspension of *Callicarpa maingayi *was yellowish-green in colour. However, after addition of AgNO_3_ and stirring for 48 h at room temperature, the emulsion turned dark brown. The colour changes in aqueous solutions are due to the surface plasmon resonance phenomenon (Figure 1a,b). The result obtained in this investigation is interesting because it can serve as a foundation in terms of identification of potential forest plants for synthesizing Ag-NPs.

*Callicarpa maingayi *as an aldehyde can reduce silver ions to Ag-NPs. The possible chemical equations for preparing the Ag-NPs are:


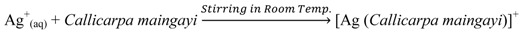
(1)


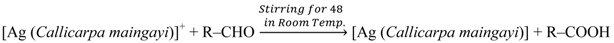
(2)

After dispersion of silver ions in the *Callicarpa maingayi *aqueous solution matrix (Equation 1), the extract was reacted with the Ag to form [Ag (*Callicarpa maingayi*)]^+^ complex, which reacted with aldehyde groups in the molecular structure of the methanolic extract to form [Ag (*Callicarpa maingayi*)] due to the reduction of silver ions through the oxidation of aldehyde to carboxylic acid groups (Equation 2).

### 2.1. UV-Visible Spectroscopy Analysis

**Figure 2:**
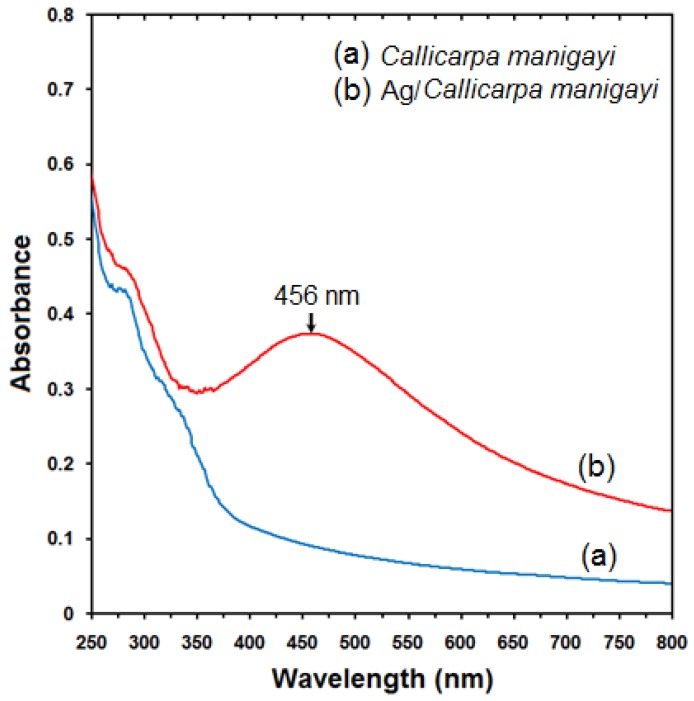
UV-Vis absorption spectra of (**a**) *Callicarpa maingayi* and (**b**) Ag/*Callicarpa maingayi *emulsion after 48 h.

The formation of Ag-NPs was followed by measuring the surface plasmon resonance (SPR) of the *Callicarpa maingayi *and Ag/*Callicarpa maingayi *emulsions over the wavelength range from 300–700 nm (Figure 2a,b). The SPR bands are influenced by the size, shape, morphology, composition and dielectric environment of the prepared nanoparticles [24,25]. Previous studies have shown that the spherical Ag-NPs contribute to the absorption bands at around 425–475 nm in the UV-visible spectra [26]. These absorption bands were assumed to correspond to the Ag-NPs extra fine nature, with relatively small size (less than 15 nm). UV-Vis absorption spectra (Figure 2b) showed that the broad SPR band contained one peak at 456 nm. This peak illustrates the presence of homogeneous distribution of hydrosol Ag-NPs after 48 h of stirring times [11,23].

### 2.2. Powder X-ray Diffraction

**Figure 3:**
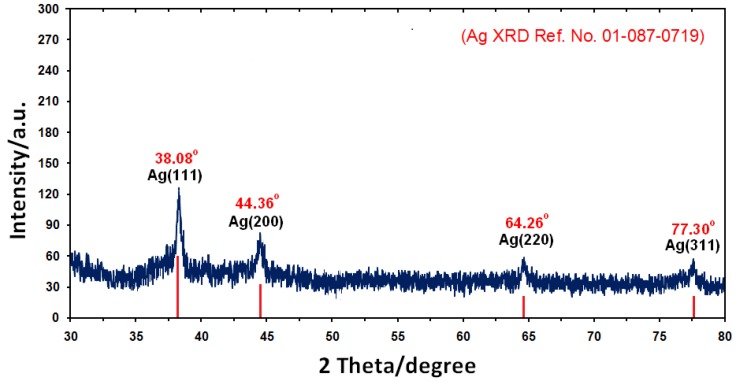
XRD patterns of Ag-NPs synthesized in *Callicarpa maingayi *for determination of Ag-NPs after 48 h.

Figure 3 shows the X-ray diffraction (XRD) patterns of vacuum-dried Ag-NPs synthesized using *Callicarpa maingayi*. The XRD patterns of Ag/*Callicarpa maingayi *indicated that the structure of Ag-NPs is face-centered cubic (fcc) [27]. In addition, all the Ag-NPs had a similar diffraction profile and XRD peaks at 2θ of 38.08°, 44.36°, 64.26°, and 77.30° could be attributed to the 111, 200, 220 and 311 crystallographic planes of the face-centered cubic (fcc) silver crystals, respectively [28]. The XRD pattern thus clearly illustrated that the Ag-NPs formed in this study are crystalline in nature. The main crystalline phase was silver and there was no obvious other phases as impurities were found in the XRD patterns (Ag XRD Ref. No. 01-087-0719).

The average particle size of Ag-NPs can be calculated using the Debye-Scherrer Equation (3):


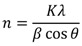
(3)

where K is the Scherrer constant with value from 0.9 to 1 (shape factor), where *λ* is the X-ray wavelength (1.5418 Å), ß1/2 is the width of the XRD peak at half height and *θ* is the Bragg angle. From the Scherrer equation the average crystallite size of Ag-NPs for sample at 48 h of stirring time are found to be lower than 15 nm in size, which are also in line with the TEM results discussed later.

### 2.3. Morphology Study

**Figure 4:**
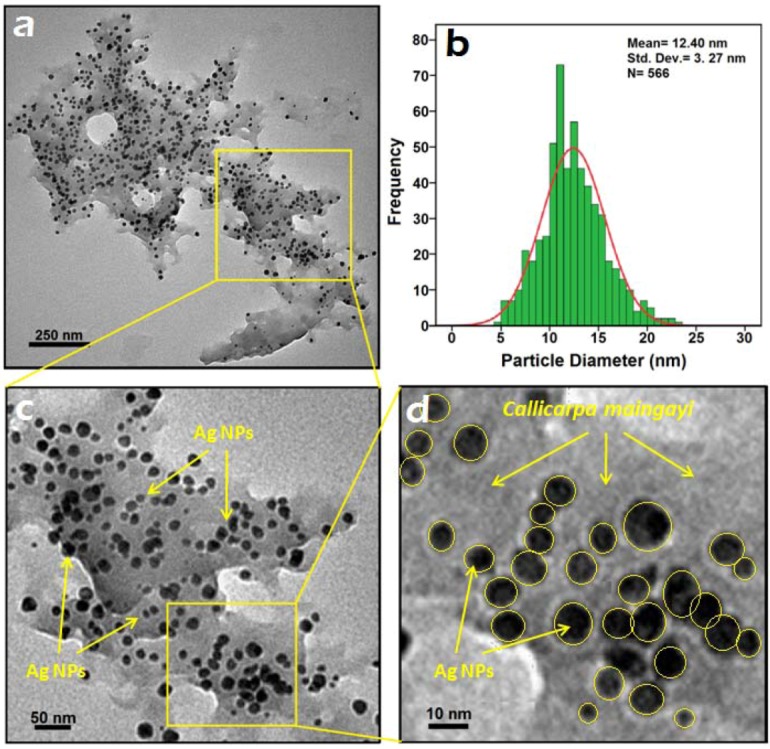
TEM image and corresponding size distribution of Ag/*Callicarpa maingayi *after 48 h.

For the transmission electron microscopy (TEM) study, a drop of the Ag-NPs solution synthesized by treating silver nitrate solution with *Callicarpa maingayi *was deposited onto a TEM copper grid. After drying, the grid was imaged using TEM. The TEM image and their size distribution are shown in Figure 4a,b, the result showed narrow particle size distributions, with diameters in the range of 9.13–15.67 nm. Moreover the mean diameter and standard deviation of Ag-NPs is 12.40 ± 3.27 nm.

**Figure 5:**
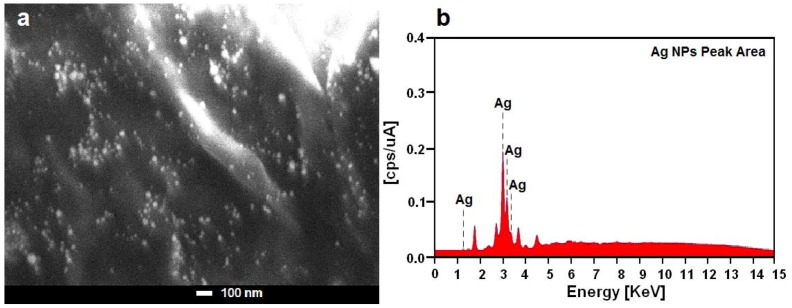
SEM image and EDXRF spectrum of Ag-NPs formed in *Callicarpa maingayi* Stem bark extraction after 48 h.

The presence of one narrow distribution of Ag-NPs in TEM image are in accordance with the UV-Vis spectral study. Figure 4c,d show the Ag-NPs surrounded by the extract of *Callicarpa maingayi*. The dark points in this figure represent the large scale distribution of Ag-NPs. The Ag-NPs surrounded by *Callicarpa maingayi* extract is shown by TEM in Figure 4 and confirmed by FT-IR spectroscopy. The numbers of Ag-NPs counted for TEM image was around 566 at 48 h stirring time. These results confirms that extract of *Callicarpa maingayi* can effectively control shape and size of the Ag NPs. Figure 5a,b show the SEM image and EDXRF spectrum for the Ag-NPs in the *Callicarpa maingayi *extraction after 48 h.

The external surfaces of Ag/*Callicarpa maingayi* gradually become more shiny, due to the presence of small size Ag-NPs (Figure 5a). A similar phenomenon was reported by *Chandra et al*. In the Figure 5b, the peaks around 1.3, 3.1, 3.3 and 3.4 keV are related to the silver elements in the *Callicarpa maingayi* [29].

Additionally, the EDXRF spectra for the Ag/*Callicarpa maingayi* confirmed the presence of Ag-NPs in the stem bark extraction without any impurity peaks. From EDAX spectrum, it is clear that *Callicarpa maingayi* has percent yield of 41.63% of Ag-NPs. The results indicate that the synthesized nanoparticles are composed of high purity Ag-NPs.

### 2.4. FT-IR Chemical Analysis

**Figure 6:**
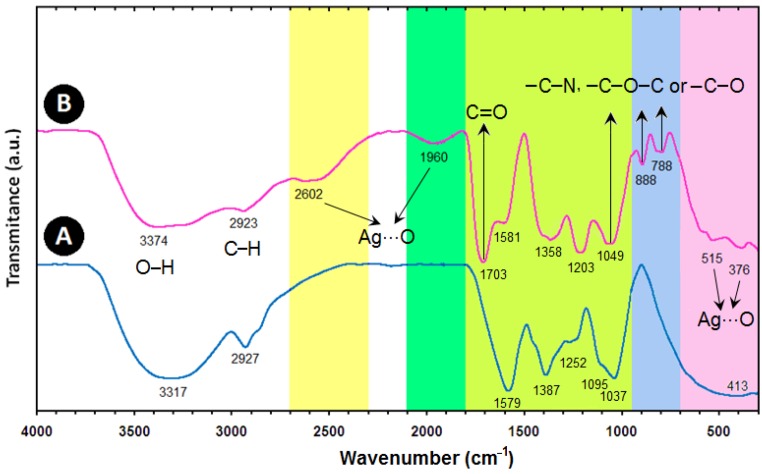
FT-IR spectra for the *Callicarpa maingayi* (**A**) and Ag/*Callicarpa maingayi* stem bark (**B**) after 48 h from biosynthesis reaction.

The FT-IR spectra were recorded to identify the possible biomolecules responsible for the reduction of the Ag^+^ ions and capping of the bioreduced Ag-NPs synthesized by the *Callicarpa maingayi *stem bark extract. The *Callicarpa maingayi *stem bark extract after complete bioreduction of Ag^+^ was centrifuged at 18,000 rpm for 20 min. to isolate the Ag-NPs from proteins and other compounds present in the solution. Figure 6A shows the FT-IR spectrum of pure *Callicarpa maingayi *stem bark extracts that did not contain AgNO_3_, where as Figure 6B shows the spectrum containing Ag-NPs after extract bioreduction with AgNO_3_. The spectrum in Figure 6A shows transmission peaks at 3317, 2927, 1579, 1387, 1252, 1095, 1037 and at 413 cm^−1^. Similarly, transmission peaks for the stem bark extract containing Ag-NPs were obtained at 3379, 2923, 2602, 1960, 1703, 1581, 1358, 1203, 1049, 888, 788, 515 and 376 cm^−1^. Three absorption peaks located around 888, 788 and 1049 cm^−1^ can be assigned as the absorption peaks of –C–N stretching vibrations of the amine, –C–O–C or –C–O groups, respectively [30]. The bonds or functional groups such as –C–O–C–, –C–O and –C=C– derived from heterocyclic compounds, e.g., alkaloid, or flavones, and the amide I bond derived from the proteins which are present in the stem bark extract are the capping ligands of the nanoparticles [31]. The broad and strong bands at 3379 to 2923 cm^−1^ were due to bonded hydroxyl (–OH) or amine groups (–NH) and aliphatic C–H of the *Callicarpa maingayi *stem bark extract, respectively. The peak at 1703 cm^−1^ is attributed to the carboxyl group (–C=O) stretching vibration. The adsorption at around 1358 cm^−1^ notably showed that –NO_3_ existed in residual amounts.

The peaks at 2602 and 1960 cm^−1^ which have high wavenumber and also 515 and 376 cm^−1^ in the low wavenumber, propose the presence of van der Waals forces of interaction between oxygen groups in protein structures and in *Callicarpa maingayi *extract on the surface of Ag-NPs (Ag…O) [11]. Therefore, the FT-IR results imply that the carboxyl (–C=O), hydroxyl (–OH) and amine (–NH) groups of *Callicarpa maingayi* stem bark extracts are mainly involved in fabrication of Ag-NPs.

### 2.5. Zeta Potential Measurement

**Figure 7:**
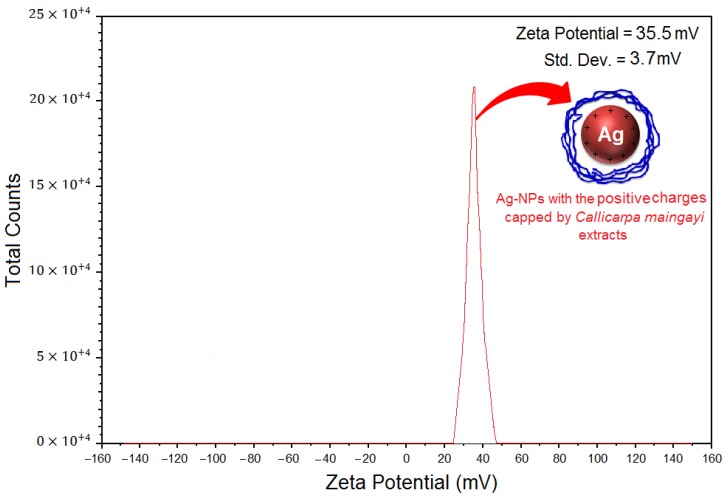
Zeta potential measurements for Ag/*Callicarpa maingayi* extract after 48 h of stirring time.

As shown in Figure 7, the Ag-NPs obtained possess a positive zeta potential value. Zeta potential is an essential parameter for the characterization of stability in aqueous nanosuspensions. A minimum of ±30 mV zeta potential values is required for indication of stable nanosuspension [32]. At 48 h of stirring time, the zeta potential was equal to 35.5 ± 3.7 mV. So, this result clearly indicated that the particles are fairly stable due to the electrostatic repulsion.

## 3. Experimental

### 3.1. Materials and Method

The stem bark of *Callicarpa maingayi* was collected from Terengganu, Malaysia. A voucher sample (No. SK 1542/08) has been deposited at the Herbarium of the Institute of Bioscience, Universiti Putra Malaysia (IBS-UPM). AgNO_3_ (99.98%) was used as a silver precursor, and was provided by Merck (Frankfurter, Germany). All reagents in this effort were analytical grade and were used as received without further purification. All solutions were freshly prepared using double distilled water and kept in the dark to avoid any photochemical reactions. All glassware used in experimental procedures were cleaned in a fresh solution of HNO_3_/HCl (3:1, v/v), washed thoroughly with double distilled water, and dried before use.

### 3.2. Extraction Preparation

**Figure 8:**
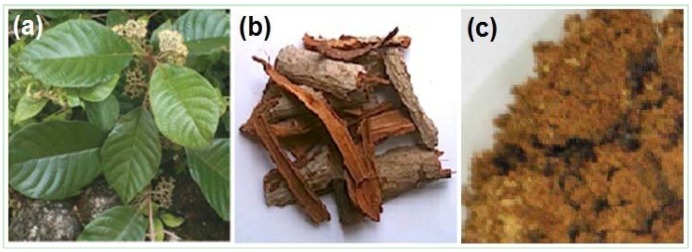
*Callicarpa maingayi* Plant (**a**); Stem bark of *Callicarpa maingayi* (**b**); Stem bark powder of *Callicarpa maingayi* (**c**).

*Callicarpa maingayi* plant and stem bark are shown in Figure 8a,b. *Callicarpa maingayi* stem bark was washed and dried in an oven dryer at 40 °C for 48 h. The stem bark were then ground into powder, stored in dark glass bottles and kept at −20 °C until further analyses Figure 7c. The finely ground *Callicarpa maingayi* stem bark (20 g) was extracted with methanol/water (ratio 80:10 v/v) at room temperature for 72 h using a shaker (Protech, Terengganu, Malaysia).

After filtration with Whitman filter paper No. 1 using a vacuum pump, the residue was re-extracted again with methanol solvent. The solvent was completely removed using a rotary vacuum evaporator (Buchi, Flavil, Switzerland) at 40 °C. The concentrated extract was then kept in dark bottles at 4 °C until used.

### 3.3. Synthesis of Ag/*Callicarpa maingayi* Emulsion

Briefly, methanolic extract of stem bark *Callicarpa maingayi* (1 g) was added to distilled de-ionized water (100 mL) with vigorous stirring for 1 h. A hundred milliliters of Ag NO_3_ (1 × 10^−2^ M) was then added and mixed at room temperature (25 °C) for 48 h. Ag-NPs were gradually obtained during the incubation period.

### 3.4. Characterization Methods and Instruments

The prepared Ag/*Callicarpa maingayi* were characterized by ultraviolet-visible spectroscopy (UV-Vis), X-ray diffraction (XRD), transmission electron microscopy (TEM), scanning electron microscopy (SEM), energy dispersive X-ray fluorescence spectrometry (EDXRF) and Fourier transform infrared (FT-IR) spectroscopy. The UV-visible spectra were recorded over the 300–700 nm range with a UV 1650 PC-Shimadzu B UV-visible spectrophotometer (Shimadzu. Osaka, Japan). The structures of the Ag-NPs produced were examined by X-ray diffraction (XRD-6000, Shimadzu). The XRD patterns were recorded at a scan speed of 4°/min. TEM observations were carried out on a H-7100 electron microscope (Hitachi, Tokyo, Japan), and the particle size distributions were determined using the UTHSCSA Image Tool version 3.00 program. SEM was performed using a Philips XL-30 instrument (Philips, Eindhoven, The Netherlands) to study the morphology of Ag/*Callicarpa maingayi*. The EDXRF was carried out on a DX-700HS spectrometer (Shimadzu). Meanwhile, the FT-IR spectra were recorded over the range of 400–4000 cm^−1^ using a FT-IR Series 100, 1650 Perkin Elmer spectrophotometer (Los Angeles, California, USA). The zeta potential measurements were also performed using a Zetasizer Nano-ZS (Malvern Instruments, Worcestershire, UK).

## 4. Conclusions

The Ag-NPs with an average size of 12.40 ± 3.27 nm and spherical in shapes were synthesized using methanolic stem bark extract of *Callicarpa maingayi*. The Ag-NPs were characterized by UV-visible, XRD, TEM, SEM, EDXRF, Zeta Potential and FT-IR spectrum. Biosynthesis of Ag-NPs using green resources like *Callicarpa maingayi* is a better alternative to chemical synthesis, since this green synthesis is pollutant free and eco-friendly. From the results obtained in this effort, one can affirm that *Callicarpa maingayi* stem bark can play an important role in the bioreduction and stabilization of silver ions to Ag-NPs.
